# Pharmacokinetics of transdermal buprenorphine patch in the elderly

**DOI:** 10.1007/s00228-012-1320-8

**Published:** 2012-06-17

**Authors:** Nabil Al-Tawil, Ingegerd Odar-Cederlöf, Anna-Carin Berggren, Helen E. Johnson, Jan Persson

**Affiliations:** 1 Karolinska Trial Alliance Phase 1 Unit, M62, Karolinska University Hospital, Stockholm, Sweden; 2 Karolinska University Hospital, Huddinge, Sweden; 3 Mundipharma AB, Göteborg, 41263 Sweden; 4 Mundipharma Research Limited, Cambridge, UK

**Keywords:** Buprenorphine, Transdermal, Patch, Pharmacokinetics, Elderly

## Abstract

**Purpose:**

Transdermal buprenorphine patches provide comparable pain relief to that of low-potency opioids in elderly individuals. However, specific data on their use in elderly individuals is limited. This study investigated and compared the PK of buprenorphine transdermal patches in elderly (≥75 years) versus younger (50–60 years) individuals.

**Methods:**

This was a multiple-dose, open-label, parallel-group study in healthy volunteers split into two age groups (younger, 50–60 years; elderly, ≥75 years) with 37 individuals in each. Study participants received two consecutive 7-day buprenorphine 5 μg/h transdermal patch applications, and blood samples were collected on the week of the second patch application [day 7 (predose), days 8, 9, 10, 12, and 14] to determine PK at steady state. Pharmacokinetic parameters were determined for buprenorphine and norbuprenorphine. Safety was assessed by analyzing adverse events, hematology, clinical chemistry, urine analysis, vital signs, electrocardiogram (ECG), and physical examinations.

**Results:**

The area under the plasma concentration-time curve at steady state (AUC_tau_), measured over one dosing interval, was similar for elderly [mean ± standard deviation (SD) 9,940 pg/h/ml (4,827 pg/h/ml] and younger [mean ± SD 11,309 (3,670 pg/h/ml] individuals. Bioequivalence was not demonstrated between groups, which may be attributable to the relatively high level of variability in individual plasma profiles. More adverse events were reported by younger (216) than elderly (164) study participants.

**Conclusions:**

No dosage alterations are necessary for PK reasons when treating elderly people with buprenorphine transdermal patches.

## Introduction

Osteoarthritis (OA) is recognized as being a common disease in the elderly population. Figures from the World Health Organization [1] indicate that in those >60 years of age, 9.6 % of men, and 18.0 % of women will have some symptoms of OA, and pain is likely to be the most troublesome symptom. Swedish guidelines [2], in common with other countries such as the UK [3], recommend paracetamol and/or nonsteroidal anti-inflammatory drugs (NSAIDs) for the initial treatment of OA pain. However, NSAIDs are not suitable in the elderly population because they increase the risk of gastric ulcers, kidney dysfunction, increased blood pressure, and heart failure [4–6]. If the initial treatment regimen does not provide adequate pain relief, second-line treatment with a low-potency opioid analgesic is suggested [2].

The availability of low-dose buprenorphine patches makes them a candidate for this second-line treatment according to these guidelines, and the transdermal route of administration is suitable in the elderly population [7]. Adverse events (AEs), common among opioids, include the risk of respiratory depression [8], suppressed immune response [9], effect on the gonadal hormones [10], and a possible contribution to the induction of hyperalgesia [11]. Buprenorphine has a ceiling effect for respiratory depression [12, 13], which confers a greater safety margin against this serious AE. Buprenorphine does not cause immunosuppression [14], has no effect on hormone levels [14], and has a pronounced antihyperalgesic effect [15]. Also, buprenorphine clearance is only dependent on renal function to a limited degree, making it appropriate for use in those with impaired renal function in whom no dose adjustment is necessary [7] [16]. Finally, in common with other opioids, if needed, its effects can be completely reversed by naloxone [17].

All these factors combined indicate that buprenorphine patches can be considered a particularly suitable method of pain relief in the elderly. A possible caveat could possibly be increased transdermal bioavailability or decreased elimination in the elderly. To investigate these concerns, this study was compared the PK (PK) of buprenorphine patches in elderly (≥ 75 years) and younger (50-60 years) individuals.

## Methods

### Overview and study design

This was a single-center, multiple-dose, open-label, parallel-group, phase 1 study conducted to characterize the PK of buprenorphine 5 μg/h transdermal patch. The low-dose strength and short treatment period (2 weeks) were decided upon in order to limit the number of AEs that might occur and to increase compliance. The study was designed and conducted in accordance with Good Clinical Practice guidelines, received ethics approval from an independent ethics committee in Stockholm, Sweden, and was conducted between 7 January 2010 (first participant first visit) and 26 March (last participant last visit) 2010 by a contract research organization [Trial Form Support (TSF) AB, Sweden] on behalf of Mundipharma AB, Sweden.

### Study participants and setting

Of the 89 participants enrolled, 74 were randomized into the study and were split into two groups depending on age (younger: 50-60 years; elderly: ≥ 75 years). There were 37 participants in each group, and a minimum of 72 (36 in each group) were required for evaluation. To enter into the study, all participants had to provide informed consent and inclusion/exclusion criteria had to be fulfilled. Inclusion criteria were men and aged 50–60 years of age in one group and ≥75 years in the other group, at a ratio of 1:1. Exclusion criteria were history of or ongoing chronic condition requiring frequent analgesic therapy (e.g., OA, frequent headaches, frequent migraine, gout, rheumatoid arthritis); individuals on ongoing opioid treatment or had been on opioid treatment during the 3 months prior to the screening visit; any recent history of frequent nausea or emesis regardless of etiology.

### Treatments

All participants received treatment with two consecutive buprenorphine 5 μg/h transdermal patches (Norspan® Mundipharma). In the first week, the patch was applied to the right upper outer arm on day 0 and was removed a week later, on day 7. The second patch was applied on day 7 on the left upper outer arm and removed on day 14. The patches were applied and removed by study-site staff. Application site was to be on nonirritated, intact skin without scars and relatively hairless, or hair was to be removed with scissors, not shaven. The area was to be clean (cleaned with water only, if needed; no soap, alcohol, oil, lotion, or abrasive devices) and dry before application. Upon application, the patch was pressed firmly in place for up to 30 s to ensure contact was complete. Should the patch start to peel at any stage during the week, it was to be stuck down with skin tape. Study participants were allowed to shower, not bathe, but had to refrain from showering until the day after the patch application and were not allowed to wash or rub the site of the patch application. Medication or substances that were not allowed, such as alcohol and monoamine oxidase inhibitors (MAOIs), were listed in the exclusion criteria. Any substance metabolized via cytochrome (CY)P3A4, including grapefruit juice, was also prohibited throughout the study period.

### Pharmacokinetic evaluations

Blood samples (6 ml each) were collected for PK analysis on day 7 (predose) and days 8, 9, 10, 12, and 14 [e.g., at 0 (predose), 24, 48, 72, 120, 168 (±2 hours)] and were analyzed under blinded conditions.

### Buprenorphine assay in plasma

A sensitive and highly specific method of determining buprenorphine and norbuprenorphine in human plasma using liquid chromatography tandem mass spectrometry (LC-MS/MS) with electrospray ionization was validated. Following solid-phase extraction using mixed-mode cation-exchange extraction plates, samples were evaporated to dryness under nitrogen at 50 °C before reconstitution in acetonitrile and injection onto the LC-MS/MS system. An ultraperformance LC (UPLC) system with a C18 1.7-μm acquity (100 × 2.1 mm i.d.) reversed-phase column was used for chromatographic separation, together with a mixture of acetonitrile and 10 mM ammonium formate buffer (pH 3) as the mobile phase. The lower limit of quantification (LLQ) was 25 pg/ml for buprenorphine and norbuprenorphine, with calibration curves linear over the range for buprenorphine (25–25,000 pg/ml) and norbuprenorphine (25–10,000 pg/ml). The mass spectrometer used was a Sciex API 5000, and nominal masses for precursor/product ions were 468/396 m/z for buprenorphine and 414/101 mass-to-change ratio (m/z) for norbuprenorphine. Buprenorphine-D4 and norbuprenorphine-D9 were used as internal standards, and recoveries were between 76 % and 82 %, respectively; intrabatch precision and accuracy were ≤ 6.1 % and ≤ ± 6.8 % for buprenorphine and ≤ 10.8 % and ≤ ± 14.0 % for norbuprenorphine. Interbatch precision and accuracy was ≤ 4.8 % and ≤ ± 4.8 % for buprenorphine and ≤ 9.5 % and ≤ ± 8.0 % for r norbuprenorphine.

Some participants had one or more buprenorphine concentration measurements below the LLQ. In order for data from these participants to be included in the analysis, and to ensure that mean parameters were not reported as being artificially high due to exclusion of these values, all plasma concentration levels of buprenorphine and norbuprenorphine below the LLQ (< 25 pg/ml) were presented as half (12.5 pg/ml) of the LLQ. This was done before PK analysis was carried out. Plasma concentrations were used to calculate the area under the plasma concentration-time curve measured from the time of dosing over one dosing interval at steady state (AUC_tau_), maximum observed plasma concentration measured over one dosing interval at steady state (C_maxss_ ), minimum observed plasma concentration measured over one dosing interval at steady state (C_minss_), and the fluctuation index (FI). AUC_tau_ was determined using the linear trapezoidal method. C_maxss_ and C_minss_ were obtained directly from reported data. FI was determined from the ratio of C_maxss_ to C_minss_. The primary PK comparison was based on the PK variable AUC_tau_ of buprenorphine at steady state. Secondary PK comparisons were based on the AUC_tau_ of norbuprenorphine and C_maxss_, C_minss_, and FI of both analytes (buprenorphine and norbuprenorphine). All PK calculations were conducted by Mundipharma Research Limited using WinNonlin Enterprise® version 4.1, Pharsight, Mountain View, CA, USA.

### Safety evaluations

During the trial, AEs were recorded, and safety was monitored continuously. Standard laboratory safety tests (hematology, clinical chemistry, urinalysis), and assessment of vital signs and electrocardiograms (ECG) were performed at screening, end of treatment (day 14), and the follow-up visit (urinalysis was not carried out at follow-up). A physical examination was carried out at screening, day 14, and the follow-up visit (day 21).

### Statistical analyses

The sample size for this study was chosen to compare ratios of AUC_tau_ between the two age groups. A previous phase 1 study (unpublished data) provided an estimated SD for the log-transformed AUC_tau_ of 0.318. Therefore, 72 participants (36 in each age group) would be needed to provide 80 % power to obtain a 90 % confidence interval (CI) for the ratio that lay between 80 % and 125 %. It was assumed there would be some withdrawals, so approximately 90 potential participants were needed to achieve 72 actual participants for evaluation. The enrolled population all provided written informed consent. The safety population had the buprenorphine patch applied, with at least one safety assessment after patch application; the full analysis set (FAS) for PK metrics all had at least one PK metric calculated. The following protocol violations excluded a participant from the FAS: failure to comply with the inclusion/exclusion criteria, significant failure to comply with the treatment regime, significant deviation from the study schedule where treatment duration or visit intervals were significantly different to the schedule in the protocol, and failure to collect data for the primary endpoint as detailed in the protocol (i.e., one or more PK blood sample was missing).

The primary objective of this study was to determine whether elderly individuals (≥75 years) have similar PKs to younger individuals (50-60 years) when treated with buprenorphine transdermal patches. For the primary PK variable (buprenorphine AUC_tau_), the following null hypothesis (H_0_) was tested versus the alternative hypothesis (H_1_): H_0_ (R < 80 %) or (R > 125 %), H_1_ 80 % ≤ R ≤ 125 % , where R = ratio of buprenorphine AUC_tau_ geometric mean levels at steady state between the elderly and the younger age populations. Log-transformed data of AUC_tau_ were modelled using linear regression with a fixed term for age group, which is identical to a simple* t* test comparing age groups. Ratios between age groups and their associated 90 % CIs were calculated by back-transforming estimates from the linear regression from the logarithmic scale to the original scale. A statistically significant result of bioequivalence between age groups was to be concluded if the 90 % CI for the ratio of mean AUC_tau_ comparing age groups lay between 80 % and 125 %. This method is equivalent to two one-sided tests at the 5 % significance level, requiring rejection of both hypotheses (R < 80 %) and (R > 125 %) to state bioequivalence. The secondary PK variables (AUC_tau_ of norbuprenorphine and C_maxss_, C_minss_, and FI of both buprenorphine and norbuprenorphine) were analyzed using the same equivalence limits and the same method as for the primary PK variable.

## Results

### Participant demographics and disposition

Eighty-nine participants were enrolled in the study, and 74 were randomized to treatment with buprenorphine patches (37 in the younger age group and 37 in the older age group). Fifteen were excluded from the randomized population because they either did not meet the inclusion criteria or they met the exclusion criteria. The safety population and the FAS were used for analysis. The safety population included all 37 participants in each group. Demographic data for all participants in the safety population is given in Table 1, which shows that there were no notable differences between groups apart from age (as required by the study) and sex. Unequal gender distribution is a factor in OA as more women than men suffer from the condition. The FAS included 36 participants in each group. One participant from each group was excluded because they deviated from the study schedule with regards to the intervals between visits; for one participant in the elderly group, the day-7 visit was carried out 1 day too early, meaning the patch was removed prematurely and assessments were carried out on the wrong day; and one participant in the younger group failed to comply with the treatment regimen. Therefore, these individuals were excluded from the FAS. Two participants did not have the full 14-day exposure to the buprenorphine patch; in addition to the aforementioned participant who removed the patch 1 day prematurely, one further participant left the study due to AEs, which were reported the day following treatment initiation. Thus, almost all participants (97.3 %) had the patches attached for the entire 2-week study duration. Compliance was not formally assessed but could be assumed on the basis of the determined plasma concentrations of buprenorphine and norbuprenorphine. Patch-wear observations were also carried out on days 7, 8, 9, 10, 12, and 14.

**Table 1:** Summary of demographic data (safety population)

	Statistic	Younger (*n* = 37)	Elderly (*n* = 37)	Total (*n* = 74)
Age (years)	Mean (SD)	53.8 (3.1)	78.7 (3.3)	66.3 (12.9)
	Min, max	50, 60	75, 91	50, 91
Sex	Male	12 (32.4%)	8 (21.6%)	20 (27.0%)
	Female	25 (67.6%)	29 (78.4%)	54 (73.0%)
Body mass index (kg/m^2^)	Mean (SD)	26.48 (3.78)	25.82 (3.97)	26.15 (3.87)
	Min, max	20.1, 35.7	18.2, 34.0	18.2, 35.7
	<18.5		1 (2.7%)	1 (1.4%)
	18.5 to <25	15 (40.5%)	18 (48.6%)	33 (44.6%)
	25 to <30	14 (37.8%)	13 (35.1%)	27 (36.5%)
	30 to <35	7 (18.9%)	5 (13.5%)	12 (16.2%)
	35 to <40	1 (2.7%)		1 (1.4%)
Cigarette smoker	No	32 (86.5%)	34 (91.9%)	66 (89.2%)
	Yes	5 (13.5%)	3 (8.1%)	8 (10.8%)

*SD* standard deviation

### Pharmacokinetic evaluations

**Fig. 1:**
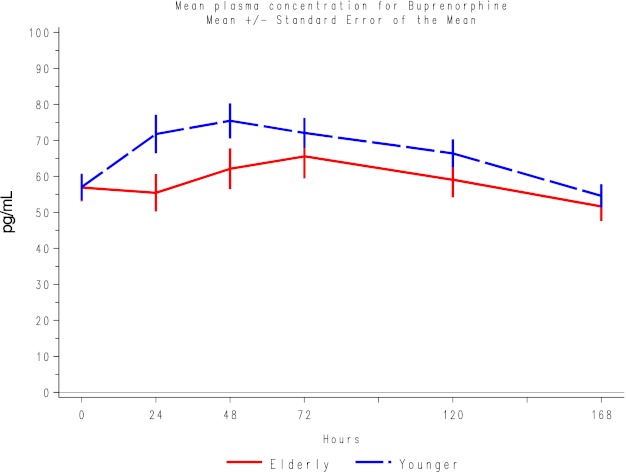
Mean plasma concentrations for buprenorphine for each group. Data points are mean ± standard error of the mean

When plasma concentrations for buprenorphine were examined, mean concentrations in the younger participants were slightly higher than in the elderly participants (Fig. 1).

**Fig. 2:**
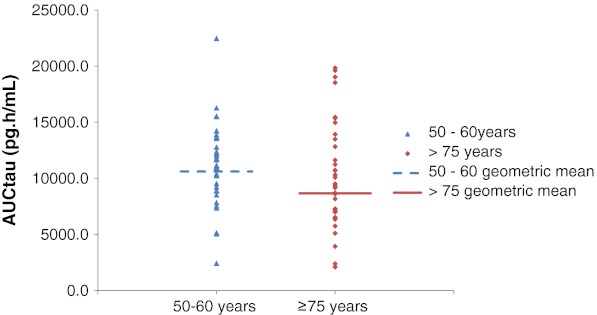
Area under the plasma concentration-time curve at steady state (AUC_tau_) buprenorphine.* Data points* are individual participant values; the* horizontal line* represents the geometric mean for each population

Regarding the primary PK variable (mean AUC_tau_ of buprenorphine) (Fig. 2), mean values at steady state were similar for younger and elderly participants, but a large interparticipant variability was observed in the elderly group, as demonstrated by the SD, standard error of the mean (SE), and ranges of the values (Table 2). The ratio (elderly/younger) of geometric mean levels at steady state was 81.6 %; the 90 % CI for the ratio was 64.7–102.9 (Table 2). The secondary PK variable results for buprenorphine showed that the mean values for C_minss_ and FI were similar for both groups; however, mean C_maxss_ values were lower in the elderly group [mean (SD) 72.15 (34–79)] than the younger group [mean (SD) 83.46 (28–97)]. Bioequivalence criteria were not met for any of the secondary PK variables for buprenorphine (Table 2). For norbuprenorphine, bioequivalence was shown for AUC_tau_ at steady state (ratio elderly/younger of the geometric mean 100.7 %; 90 % CI 82.8–122.4). Mean C_maxss_ were also similar for both groups, but bioequivalence was not shown, perhaps due to the large variability in the results. For the remaining variables, C_minss_ and FI values were slightly higher, longer, and lower, respectively, for elderly participants compared with younger participants, and bioequivalence was also not shown for these parameters (Table 2).

**Table 2:** Pharmacokinetic variables for buprenorphine and norbuprenorphine (FAS)

Variable	Statistic	Buprenorphine		Norbuprenorphine	
		Younger (*n* = 36)	Elderly (*n* = 36)	Younger (*n* = 36)	Elderly (*n* = 36)
AUC_tau_ (pg/h/ml)	No. of observations	36	36	36	36
	Mean (SD)	11,309.33 (3,669.51)	9,939.87 (4827.13)	2,922.00 (1338.08)	3,063.23 (1,771.45)
	Min, max	2,421.6, 22464.0	2,100.0, 19842.0	2,100.0, 6631.2	2,100.0, 7669.2
C_maxss_ (pg/ml)	No. of observations	36	36	36	36
	Mean (SD)	83.46 (28.97)	72.15 (34.79)	21.73 (12.48)	20.46 (13.17)
	Min, Max	25.9, 169.0	12.5, 153.0	12.5, 50.0	12.5, 56.5
C_minss_ (pg/ml)	No. of observations	36	36	36	36
	Mean (SD)	47.71 (18.17)	47.45 (22.89)	13.85 (4.59)	16.33 (8.21)
	Min, max	12.5, 110.0	12.5, 93.9	12.5, 31.7	12.5, 42.7
Fluctuation index	No. of observations	36	36	36	36
	Mean (SD)	1.89 (0.72)	1.61 (0.73)	1.57 (0.82)	1.22 (0.45)
	Min, max	1.2, 4.3	1.0, 5.3	1.0, 3.5	1.0, 2.6

*FAS* full analysis set,* AUC*_*tau*_ area under the plasma concentration-time curve at steady state,* C*_*maxss*_ maximum observed plasma concentration measured over one dosing interval at steady state ,* C*_*minss*_ minimum observed plasma concentration measured over one dosing interval at steady state,* SD* standard deviation,* Min, max* minimum, maximum

Most participants, regardless of age group, had one or more norbuprenorphine concentration measurements below the LLQ. In addition, two participants in each group had one or more buprenorphine concentration measurements below the LLQ. Three participants in the elderly group had no measurable plasma levels of buprenorphine during the second patch application.

### Safety evaluations

Overall, there were 380 AEs (including 264 unique AEs) in 64 study participants(86.5 %). More AEs were reported for younger (216in 35) than for elderly (164 in 29) participants (Table 3). All except four AEs were treatment related (the investigator assessed AEs as being definitely, probably, possibly, or unlikely to be related to study medication). The most common AEs in both groups were nausea, constipation, fatigue, dizziness, and headache, and all were reported by more participants in younger than in the elderly group. Most AEs were mild (250) or moderate (111) in severity; there were only 19 severe AEs, which were generally typical of the most commonly reported AEs and mostly reported by younger participants. No serious AEs were reported, but one participant in the younger group discontinued the study due to nausea and vomiting. The AEs seen during this study were consistent with the expected AE profile of opioid analgesics.

**Table 3:** Summary of adverse events (AEs); safety population

	Younger (*n* = 37)	Elderly (*n* = 37)	Total (*n* = 74)
Total number of unique AEs^a^	147	117	264
Total number of AEs	216	164	380
Total number of participants with at least one AE	35 (94.6%)	29 (78.4%)	64 (86.5%)
Total number of SAEs	0	0	0
Total number of unique related AEs ^a^	146	116	262
Total number of related AEs	213	163	376
Total number of participants with at least one related AE	34 (91.9%)	29 (78.4%)	63 (85.1%)
Total number of participants with at least one AE leading to discontinuation	1 (2.7%)	0	1 (1.4%)

^a^ AE (preferred term) counted only once for each participant, even if it occurred more than once

Generally, no abnormalities in laboratory safety, vital signs, or ECG results were seen during this study, but there was a transient increase in liver enzymes for two participants in the younger age group, which returned to normal by day 21; and one elderly participant had clinically significant high blood pressure values on day 14 and day 21, which was reported as an AE of hypertension (unlikely to be related to study medication).

## Discussion and conclusions

This is the first study examining the PKs of buprenorphine patches specifically in the elderly (>75 years). It was important to investigate the PKs of buprenorphine in this population due to the fact that the elderly often have altered pharmacodynamics (PD) and PK because of their age, and also, perhaps, due to other conditions that are more prevalent in the elderly population. A first step in studying the possible difference in effects across the age range is to determine PKs, thereby allowing the PK effects to be teased out from the influence of PDs.

This study determined that the average buprenorphine exposure at steady state is only slightly lower for the elderly than for the younger population However, a relatively high level of variability was noted in individual plasma profiles, and the 90 % CIs did not meet the 80–125 % bioequivalence criteria. Similarly, bioequivalence was not demonstrated for secondary buprenorphine and norbuprenorphine PK parameters, except for AUC_tau_ at steady state for norbuprenorphine. Again, the ratio of elderly to younger values was relatively close to 1 for all parameters (in the range 80–112 %), but confidence intervals were wide due to large interparticipant variability. More AEs were reported by younger individuals (216 AEs) than elderly participants (164 AEs). The reason for this is at present unknown but may be due to levels of exposure, as buprenorphine concentrations were, in general, higher in the blood of the younger participants. All except four AEs were treatment related.

Though bioequivalence was not formally proven between elderly and younger participants in this study, the systemic exposure to buprenorphine was sufficiently similar between groups, particularly given the large interindividual differences, which are not to be a decisive dosing factor in the clinic. There is an on-going study to evaluate the efficacy and safety of buprenorphine patches between elderly and younger populations in a clinical setting (EudraCT No. 2010-020748-37), but other studies have also examined buprenorphine use in the elderly. Likar et al. [18] carried out a study of buprenorphine patches in patients with OA who were ≥65 years and compared results with patients <65 years. The patches were found to be at least as effective in the elderly group in relieving pain, with no age-related differences in safety and with comparable plasma concentrations. The results seen by Likar et al.[18] were similar to those seen for PK and safety seen in another phase 1 study with buprenorphine patches [19]. Also, a long-term (6-month) study of buprenorphine patches versus placebo in OA patients >40 years (average approximately 63 years) previously naïve to opioids was carried out by Breivik et al. [20]. Though the primary objective was not statistically significant (24-h Western Ontario and McMaster Osteoarthritis Index of pain), the addition of buprenorphine patches (5–20 μg) provided greater relief of daytime movement-related pain compared with NSAIDs or Cox-2 selective inhibitors plus paracetamol. The impression of participants’ improvement at the end of 6 months was also significantly better with the addition of buprenorphine. Increasing age induces various changes in physiology, with changes to the skin, including reduced dermal absorption, compromised vascular response [21], and changes to hydration and lipidic structure. However, there is little evidence to suggest significant differences in transdermal absorption in the elderly [22].

Although there were a high number of AEs in our study, this was not unexpected, and the events were consistent with the expected AE profile of opioid analgesics. Elderly individuals tolerated the patches at least as well as the younger individuals. AEs were generally mild or moderate, and this study, along with others, demonstrated good adherence to buprenorphine patch treatment compared with other opioids [23], indicating a net gain of pain relief versus occurrence of any AEs and pointing toward a positive risk–benefit ratio.

## Conclusions

Although we did not prove bioequivalence of PK parameters between younger and elderly individuals, this study may suggest that no dosage alterations are necessary for PK reasons when treating elderly people with buprenorphine transdermal patches.
